# "NeuroStem Chip": a novel highly specialized tool to study neural differentiation pathways in human stem cells

**DOI:** 10.1186/1471-2164-8-46

**Published:** 2007-02-08

**Authors:** Sergey V Anisimov, Nicolaj S Christophersen, Ana S Correia, Jia-Yi Li, Patrik Brundin

**Affiliations:** 1 Neuronal Survival Unit, Wallenberg Neuroscience Center, Lund University, 221 84 Lund, Sweden

## Abstract

**Background:**

Human stem cells are viewed as a possible source of neurons for a cell-based therapy of neurodegenerative disorders, such as Parkinson's disease. Several protocols that generate different types of neurons from human stem cells (hSCs) have been developed. Nevertheless, the cellular mechanisms that underlie the development of neurons *in vitro *as they are subjected to the specific differentiation protocols are often poorly understood.

**Results:**

We have designed a focused DNA (oligonucleotide-based) large-scale microarray platform (named "NeuroStem Chip") and used it to study gene expression patterns in hSCs as they differentiate into neurons. We have selected genes that are relevant to cells (i) being stem cells, (ii) becoming neurons, and (iii) being neurons. The NeuroStem Chip has over 1,300 pre-selected gene targets and multiple controls spotted in quadruplicates (~46,000 spots total). In this study, we present the NeuroStem Chip in detail and describe the special advantages it offers to the fields of experimental neurology and stem cell biology. To illustrate the utility of NeuroStem Chip platform, we have characterized an undifferentiated population of pluripotent human embryonic stem cells (hESCs, cell line SA02). In addition, we have performed a comparative gene expression analysis of those cells versus a heterogeneous population of hESC-derived cells committed towards neuronal/dopaminergic differentiation pathway by co-culturing with PA6 stromal cells for 16 days and containing a few tyrosine hydroxylase-positive dopaminergic neurons.

**Conclusion:**

We characterized the gene expression profiles of undifferentiated and dopaminergic lineage-committed hESC-derived cells using a highly focused custom microarray platform (NeuroStem Chip) that can become an important research tool in human stem cell biology. We propose that the areas of application for NeuroStem microarray platform could be the following: (i) characterization of the expression of established, pre-selected gene targets in hSC lines, including newly derived ones, (ii) longitudinal quality control for maintained hSC populations, (iii) following gene expression changes during differentiation under defined cell culture conditions, and (iv) confirming the success of differentiation into specific neuronal subtypes.

## Background

Modern DNA microarrays permit a comprehensive analysis of quantitative and qualitative changes in RNA transcript abundance, outlining the cross-sections of gene expression and alterations of these in response to genetic or environmental stimuli. Genome-scale microarrays (cDNA- or oligonucleotide-based) are most valuable when screening populations of cells for the novel genes reflecting potential diagnostic and prognostic markers or for an identification of novel therapeutic targets. On the other hand, custom microarray platforms that focus on specific pre-selected subset of genes relevant to a particular field of investigation can be less costly and more suitable for detection of smaller gene expression changes.

Microarray technology has added important information on both normal development and pathological changes in neurons. This is well illustrated by multiple studies on *substantia nigra *dopaminergic neurons, which degenerate in Parkinson's disease (PD) [1-5]. The shortcomings of pharmacological therapies in PD have stimulated a search for alternative treatment strategies. In successful cases, transplants of human embryonic mesencephalic dopaminergic neurons can both restore dopaminergic neurotransmission and provide some symptomatic relief [6-8]. A wider application of neural transplantation in PD is, however, currently not feasible due to the unpredictable and variable outcome, the risks of unwanted side-effects (dyskinesias) [9,10] and ethical and practical problems associated with using donor cells obtained from aborted embryos and fetuses [11,12]. Human embryonic stem cells (hESCs) are considered a promising future source of cells for cell replacement therapy in PD and other neurological conditions [13]. They could constitute a virtually infinite source of self-renewing cells that can be persuaded to differentiate into specific types of neural cells, including dopaminergic neurons [14-16]. The molecular mechanisms that govern development of cultured hESCs into specific types of neural cells are not fully understood. To promote our understanding of such mechanisms, it would be valuable to have tools that readily and reproducibly can help to characterize the cells as they differentiate from pluripotent stem cells into post-mitotic neurons. This important issue was addressed in earlier studies by Luo et al. and Yang et al., who designed small-to-moderate scale custom microarray platforms (281 and 755 gene targets, respectively) [17,18]. In addition SuperArray Bioscience Corporation (Frederick, MD, USA) have manufactured a range of small-scale arrays (263 gene targets for human array; [19]). We sought to create an improved and updated microarray platform for hESC/neuronal differentiation-oriented gene expression studies. Therefore, we generated a specialized large-scale DNA microarray platform (the "NeuroStem Chip") that has over 1,300 pre-selected gene targets and multiple controls spotted in quadruplicates (~46,000 spots total). Here we introduce the platform and the advantages it can offers to neuroscientists and stem cell biologists: particularly, in the niche of gene expression-oriented characterization of the samples using an assay of pre-selected, already established gene targets. In the current study, we use the NeuroStem Chip to characterize an undifferentiated population of pluripotent hESCs (cell line SA02, Cellartis AB, Göteborg, Sweden) and compare the gene expression in those cells with that of a hESC-derived cell population rich in neurons, including tyrosine hydroxylase-positive dopaminergic neurons.

## Results and Discussion

Stem cells have unique biological characteristics, but only a limited number of genes are currently recognized as established stem cell markers. Examples include POU domain, class 5, transcription factor 1 (Oct3/4), signal transducer and activator of transcription 3 (Stat3), teratocarcinoma-derived growth factor (Tdgf1), Enk-pending (Nanog), undifferentiated embryonic cell transcription factor 1 (Utf1) and DNA methyltransferase 3B (Dnmt3b) [20]. At the same time, hundreds of genes are suggested as candidate markers for "stemness", but their coupling to the undifferentiated stem cell state is not yet fully verified [21]. The concept of "stemness" (term introduced in 1986 by Grossman & Levine) is defined as *"core stem cell properties that underlie self-renewal and the ability to generate differentiated progeny" *[22]. Considering the complexity of the processes involved, stemness can hardly be ensured by co-operation of just a few genes. Nevertheless, three stemness genes (namely, Oct3/4, Stat3 and Nanog) are considered "master"-genes that control the self-renewing process [23,24]. Various types of stem cells, such as hematopoietic, mesenchymal and neural (HSCs, MSCs and NSCs, respectively), embryonic germ and embryonic carcinoma cells (EGCs and ECCs, respectively) are all characterized by variations in gene expression profiles, and only a few gene markers are associated with all these cell types [25,26]. We have aimed to embrace the most comprehensive set of those genes into a solitary array, the NeuroStem Chip. Thereby, it is possible to employ it to monitor the relative expression levels of numerous known and candidate stemness genes in a single experiment.

Similar to the genetic bases underlying stemness, cell differentiation is associated with altered expression levels of certain recognized or candidate genes [25]. We therefore incorporated gene markers of development and differentiation in general, and that of neuronal and dopaminergic differentiation in particular, into the NeuroStem Chip. Examples include markers for the processes of neuronal maturation, axonal branching, neural/neuronal survival, etc. Finally, we ensured that known markers for specific types of neurons, allowing identification of individual cell types, were present on the chip. We paid special attention to genes associated with the differentiation and maturation of dopaminergic neurons. In many published studies, the expression of only a single (tyrosine hydroxylase, TH) or 2–3 markers for dopaminergic neurons (e.g. amino acid decarboxylase (AADC), dopamine transporter (DAT), vesicular monoamine transporter 2 (VMAT2)) have been used to indicate dopaminergic identity of neurons. In contrast, the NeuroStem Chip includes oligonucleotide probes for 88 genes related to dopaminergic neurons, thus being more comprehensive in this sense, compared to other existing microarray platforms, including focused ones [17,18]. Those entries encompass recognized and candidate markers for dopaminergic neurons (mature and early) and progenitors, as well as markers for the maturation and differentiation of the latter (Table 1).

Table 2 represents conditional functional breakdown of genes targeted by the NeuroStem microarray platform. A number of important gene groups that are included in the chip are not mentioned in Table 2. Among these, entries related to Dickkopf gene family, galanin-, melatonin-, vasoactive intestinal peptide (VIP)-, cAMP response element-binding protein (CREB)- and B cell leukemia 2 (Bcl2) oncogene-related are present. Many of them play potentially important, yet undefined, roles in the biology of stem cells. Additionally, we included some genes implicated in disease mechanisms of neurodegenerative disorders (most importantly, Parkinson's disease and Alzheimer's disease) in the chip. Furthermore, we incorporated a number of markers for distinct differentiation pathways (e.g. hematopoietic and pancreatic) and cell types (e.g. cancer subtypes and a range of normal cell types) to serve as essential controls. Taken together, we believe that in its present form NeuroStem Chip represents currently most comprehensive gene expression platform for studies on stem cells, neural/neuronal differentiation, human neurodegeneration and neuronal survival, both *in vivo *and *in vitro*. The complete layout of NeuroStem Chip will be disclosed to the academic community, upon request.

**Figure 1:**
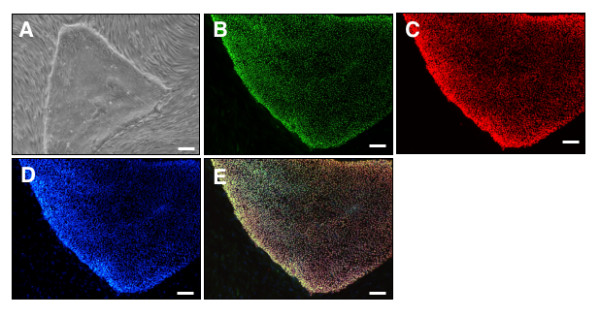
Human Embryonic Stem Cells (hESCs) used in a study. (A) Phase contrast image of unaltered hESC colony. Immunocytochemical analysis of (B) Ki67, (C) OCT3/4, (D)DAPI, (E) Merge. Scale bars = 100 μm.

**Figure 2:**
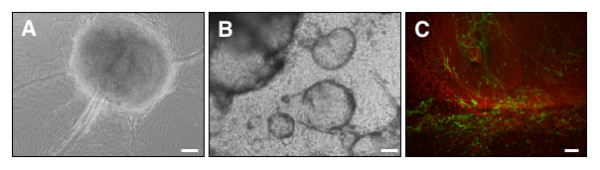
Human Embryonic Stem Cell (hESCs)-derived cells committed toward neuronal/dopaminergic differentiation pathway by co-culturing with PA6 stromal cells for 16 days. (A, B) Phase contrast images of structures formed in hESC colonies. (C) Immunocytochemical analysis of cell composition: tyrosine hydroxylase (TH), green; human nuclei marker, red. Scale bars = 100 μm.

**Figure 3:**
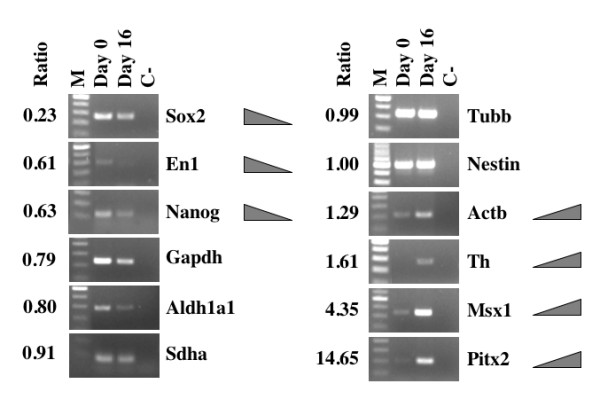
RT-PCR analysis of RNA samples used and validation of microarray results. Ratio, ratio of differentiated (Day 16)/undifferentiated (Day 0) hESC sample normalized spot intensity as detected by microarray analysis (average value from all spots). M, 100 bp DNA ladder; Day 0, undifferentiated hESCs; Day 16, hESCs committed to neuronal/dopaminergic differentiation pathway by co-culturing with PA6 stromal cells for 16 days; C-, No template control. Sox2, SRY-box 2; En1, Engrailed 1; Gapdh, glyceraldehydes-3-phosphate dehydrogenase; Aldh1a1, aldehyde dehydrogenase 1 family, member A1; Sdha, succinate dehydrogenase 2, flavoprotein sububit; Tubb, β-tubulin; Actb, β-actin; Th, tyrosine hydroxylase; Msx1, homolog of *Drosophila *muscle segment homolog 1; Pitx2, paired-like homeodomain transcription factor 2.

The microarray format we selected relies on long oligonucleotide molecules (69–71 nucleotides) printed over a solid surface. We spotted the synthesized oligonucleotides (Operon Biotechnologies) with a constant concentration across the slides, and evaluated the quality and consistency of spotting in a series of control experiments. We then illustrated the utility and technical reliability of the NeuroStem Chip by characterizing the gene expression profile of commonly utilized hESC line SA02 (Sahlgrenska 2; [27]), including (i) undifferentiated cells and (ii) cells committed towards neuronal/dopaminergic differentiation pathway. For the first of these, we used total RNA sample purified from hESC colonies that exhibited morphology consistent with cell proliferation and the absence of spontaneous differentiation (Figure 1A). We also evaluated the expression of the cell cycle marker Ki67 and the pluripotency marker OCT3/4 in the sample by immunocytochemistry (Figure 1B–E). Co-culturing of ESCs with murine stromal cells (including PA6 cell line) rapidly generates dopaminergic neurons from ESCs by an unexplained mechanism termed stromal cell-derived inducing activity (SDIA; [28,29]). We therefore committed hESCs toward the neuronal/dopaminergic differentiation pathway by co-culturing with PA6 cells for 16 days, resulting in appearance of cells positive for early and late neuronal markers, including nestin, β-III-tubulin, and TH, the established marker of dopaminergic neurons (Figure 2). To verify the expression of some key stem cell- and neural phenotype-associated genes we performed RT-PCR comparing RNA samples from the undifferentiated hESCs with hESCs of the same line differentiated toward neuronal/dopaminergic pathway, as described above. The expression profile outlined by RT-PCR confirmed the identity of the sample used (Figure 3). After performing RNA integrity tests, we incorporated fluorescent labels to the amplified RNA samples from hESCs (Cyanine 3-CTP (Cy3) and Cyanine 5-CTP (Cy5)), hESC-derived cells containing TH-positive neurons (Cy3 and Cy5) and human universal reference RNA (Cy5), and hybridized aliquots with NeuroStem microarray slides using the following conditions: hESC *vs*. reference, Cy3 : Cy5 = (i) 20:10 pmol, and (ii) 10:5 pmol; and hESC *vs*. hESC-derived cells, Cy3 : Cy5 = (iii) 30:20 pmol, respectively. Universal reference RNA has been previously established as a standard reference material for microarray experiments, proving an ability to effectively hybridize to a large fraction of microarray spots [30].

**Figure 4:**
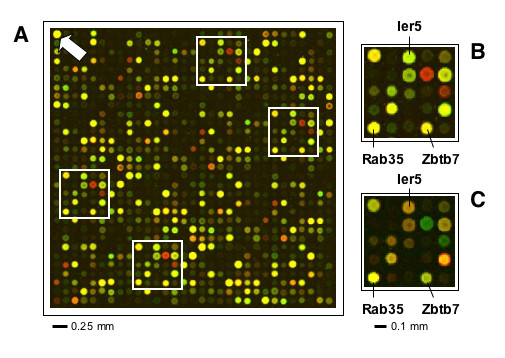
NeuroStem Chip layout (a fragment). (A) Representative block (31 × 31 spots), as hybridized with actual hESC (Cy3)/universal reference (Cy5) sample. Arrow indicates β-actin (Actb) spot, which serves as a control for grid orientation; white boxes (B) highlight repetitive patterns, illustrating quadriplication of individual spots. (B, C) Dye-swap (hESC (Cy5)/universal reference (Cy3)) indicates accuracy of fluorescent dye incorporation and hybridization chemistry. Ier5, Immediate early response 5; Rab35, RAS-associated protein RAB35; Zbtb7, Zink finger and BTB domain containing 7. Spot-to-spot center distance 140 μm; average spot size 90–110 μm.

**Figure 5:**
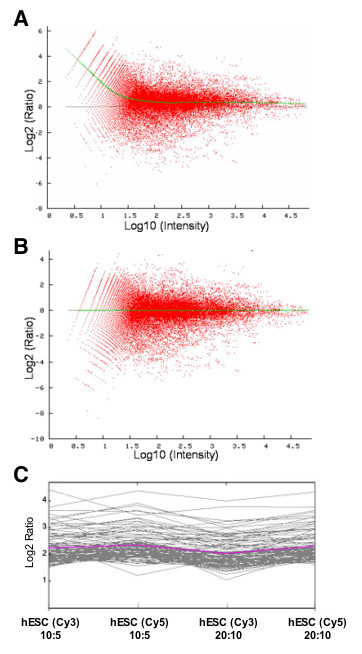
Normalization and reproducibility of the NeuroStem Chip arrays. (A-B) Representative plots depict Log_2_(ratio) versus log_10_(intensities) prior to (A) and following (B) the normalization of one technical replicate (*hESCs (Cy3) : universal reference (Cy5), 10:5 pmol*). Green lines represent fitted values after normalization. (C) Centroid graph of a K-means classifier of 101 genes clustered as highly up-regulated in the 4 technical replicates, indicating high reproducibility. Pink line is an average of the Log2 ratio values of the 101 genes. See [Additional file 1] for a list of genes in this cluster.

We performed two-color hybridizations (e.g. for the experiment *vs*. reference) following an established protocol [31], and included dye-flip technical replicates in the analysis (Figure 4). Using the online software program BASE [32] we sequentially filtered the data by background subtraction, negative flagging, negative intensities and for inconsistent data amongst replicates [33]. Figure 5A shows a comparison of the spot intensities prior to normalization (M versus A plot), with the Log2 of the expression ratio between Cy3/Cy5 being plotted as a function of the log10 of the mean of the total expression intensities for Cy3 and Cy5 channels. The deviation of the line from zero revealed a need for normalization, so prior to data analyses we normalized signals using a locally weighted scatterplot-smoothing regression (LOWESS) algorithm (Figure 5A–B; fitted line) implemented in BASE. Since the reproducibility of two-color microarray gene expression data is critically important, we calculated Pearson correlation coefficients of the reporters present in the filtered database comparing the average expression ratios (7005 for hESCs vs. universal reference; 6947 for undifferentiated vs. neuronal/dopaminergic lineage-committed hESCs). Results obtained revealed that data were consistent across technical replicates (dye-swap and amount of loaded material), showing general high reproducibility: e.g., correlation coefficients were greater than 0.96 for technical replicates and 0.78 for dye-swapping samples in hESCs vs. universal reference hybridizations (Table 3). To detect genes with high expression levels in hESC samples, we filtered data for intensity values >100 in the hESC sample and performed clustering analysis using the TIGR MultiExperiment Viewer (MEV; [34]). To visualize variations of spot/reporter per technical replicate, hierarchical clustering was performed by K-means classifier based on the linear-correlation-based distance (Pearson, centred) method. The optimal number of clusters was determined empirically to produce the most balanced ratio of entries to cluster of highly expressed genes. A cluster of 101 genes up-regulated in the hESC sample [see Additional file 1], was plotted in a centroid graph (Figure 5C); the variation across technical replicates was low. We merged technical replicates to generate a list of the most up-regulated genes expressed in the hESC sample compared to the universal reference RNA (Table 4). Standard error of the mean expressed as percentage was calculated for the 4 technical replicates, and was 6.7% for the top 25 genes up-regulated in hESC samples, compared to universal reference RNA. We performed the analysis of microarray data, as described in the Methods, and spot error values were generally in the lower range, indicating high stringency of the signals and low variance.

As seen in Table 4 and Table 5, the NeuroStem Chip identified numerous genes associated with stem cells. In particular, homeo box expressed in ES cells 1 (Hesx1) gene was identified as the most up-regulated in the ES cell preparation, compared to universal reference RNA. Highly expressed in pluripotent ESCs, Hesx1 expression is down-regulated upon embryonic stem cell differentiation [35,36], as also clearly seen in differentiation experiment of our own (Table 4). Similarly, Gremlin 1 homolog, cysteine knot superfamily gene (Grem1, also known as Cktsf1b1 and Dand2) is a recognized factor of cell-fate determination of ESCs [37]. Many more genes highly up-regulated in the hESC sample in comparison with universal reference RNA are associated with stem cells: further examples include Gap junction protein α1 (Gja1) and Zic family member 3 heterotaxy 1 (Zic3) (Table 4) [20]. The expression of fibroblast growth factor receptor 2 (Fgfr2) is of particular interest. Basic fibroblast growth factor (FGF2, bFGF) supports hESC proliferation and their ability to maintain undifferentiated phenotype when cultured *in vitro *[38,39]. Moreover, in some hESC lines a very high concentration of FGF2 could substitute for the need of feeder cells [40]. At the same time, genes listed in Table 4 represent the most highly up-regulated entries in a relatively limited group of genes (Figure 5C). Many other genes involved in maintenance of ESC phenotype (i.e. established or candidate markers of stem cells) have lower levels of expression (Table 5). Examples include undifferentiated embryonic cell transcription factor 1 (Utf1), DNA methyltransferase 3B (Dnmt3b), developmental pluripotency associated 4 (Dppa4, a newly established pluripotency marker [41]) and numerous candidate markers of "stemness": e.g. genes for KIAA1573 protein, forkhead box O1A (Foxo1a), high-mobility group box 1 (Hmgb1), C-terminal binding protein 2 (Ctbp2) and left-right determination factor 1 (Lefty1), as well as others. For numerous established or candidate markers of stem cells the expression levels were not considerably higher (Log2 ratio < 1) in the hESC sample compared to the universal reference RNA. For example, the expression of Nanog, DNA (cytosine-5-)-methyltransferase 3α (Dnmt3a), MutS homolog 2, colon cancer, nonpolyposis type 1 (E. coli) (Msh2), Thy-1 cell surface antigen (Thy1), high-mobility group box 2 (Hmgb2), transcription factor 3 (Tcf3), Nanos homolog 1 (Nanos1), MyoD family inhibitor (Mdfi), Calumenin (Calu) and soluble thymidine kinase 1 (Tk1) was detected in hES SA02 cells with Log2 ratio value < 1. Expression levels of those genes range from being inconsiderably higher to nearly equal to that in universal reference RNA sample. We believe that those findings could be explained by cellular composition of human universal reference RNA sample [42], which includes pooled RNA samples from proliferating cells (e.g., skin and testis cell lines). Thus, the relative difference between gene expression of certain markers of stem cells in undifferentiated hESCs and universal reference RNA is naturally decreased. Taken together, the gene expression signature of hES SA02 cell line profiled by NeuroStem Chip is indeed characteristic for pluripotent stem cells, providing proof-of-concept.

Notably, comparison of expression profiles of undifferentiated hESCs and hESC-derived cells committed toward dopaminergic differentiation pathway by co-culturing with SDIA for 16 days have revealed that many of the stem cell marker genes mentioned above were down-regulated in differentiation (Table 5). Expectedly, Hesx1, Grem1, Dnmt3b, Utf1 and Nanog could be listed among these. At the same time, numerous other genes, including Pitx2, Dlk1 and Msx1 were up-regulated in the latter sample ([see Additional file 2], Figure 3). Table 1 lists 24 dopaminergic system-related entries (e.g., Ptx3, Th, Lhx1) with gene expression up-regulated by Day 16 of hESC differentiation protocol; few more genes have demonstrated less prominent up-regulation (Log2 ratio values in the range of 0.7/0.97–1.0). The gene expression profiles generated are therefore consistent with the results of earlier studies utilizing hSC-derived samples with similar characteristics [43,44]. Diversity of NeuroStem Chip entries responsive to hESC commitment toward neuronal/dopaminergic differentiation pathway clearly illustrates the complexity of that pathway. The cell population obtained after 16 day exposure to SDIA is highly heterogeneous. Only around 0.2% of the cells are TH-positive cells (Figure 2). This heterogeneity, with an apparent presence of residual pluripotent cells explains the presence of stem cell marker genes, including homeobox transcription factor Nanog, as revealed by RT-PCR data (Figure 3). It would be therefore impossible to apply the platform to identify novel genes associated with the process of differentiation; for that application, the genome-scale microarray platforms (e.g., Affymetrix) are clearly superior. Nevertheless, being based upon a moderate assay of pre-selected specific gene targets, the comparative analysis of microarray data derived from undifferentiated and dopaminergic differentiate pathway-committed hESCs provides a valuable cross-cut of complex relationship between factors driving or indicative to neuronal/dopaminergic differentiation [see Additional file 2].

RT-PCR analyses have validated the overall reliability of NeuroStem microarray platform: all of the entries detected in the hybridization experiments have demonstrated similar trends when analyzed by RT-PCR means (Figure 3). Those entries include Sox2, En1 and Nanog (ratio of differentiated/undifferentiated hESC sample normalized spot intensity < 0.75, down-regulated), Gadph, Aldh1a1, Sdha, Tubb and Nestin (ratio .1.0, unchanged), Actb, Th, Msx1 and Pitx2 (ratio >1.25, up-regulated). Some of the housekeeping genes (Gapdh, Sdha, Tubb, Actb) have somewhat different expression in undifferentiated vs. differentiated cells, consistent with previous reports on certain established housekeeping genes (including Gapdh) being variable in human samples [45]. Importantly, all the observed gene expression trends were similar in both microarray and RT-PCR. Our experiment therefore confirms that the NeuroStem Chip microarray platform can still identify gene expression changes related to early stages of differentiation of hESC into dopaminergic neurons.

## Conclusion

Recent technological advances have led to DNA microarrays which contain over hundred thousand of spots of DNA material, reaching a truly genomic scale. Highly specialized DNA microarrays of smaller scale (e.g. the NeuroStem Chip) still have an important role in the directed studies in particular fields. Since they are significantly less expensive, compared to many recognized large-scale platforms (e.g. Affymetrix Human Genome platforms), they have a clear advantage in routine work involving samples from, e.g., multiple cell culture conditions. While there is a risk that one will miss out on changes in genes previously not believed to be relevant to neural differentiation, the restricted number of genes in the NeuroStem Chip also simplifies analysis and adds power. NeuroStem Chip is comparable to other stem cell-related focused microarray platforms in regards to manufacturing costs and technical simplicity of the recommended hybridization protocols. At the same time, it currently implies an advantage in both the scale and the spectrum of pre-selected, specific gene targets assayed. Some suggested areas of application for NeuroStem microarray platform could be the following: (i) characterization of the expression of established, pre-selected gene targets in human stem cell (hSC) lines, including newly derived ones, (ii) longitudinal quality control for maintained hSC populations, (iii) following gene expression changes during differentiation under defined cell culture conditions, and (iv) confirming the success of differentiation into specific neuronal subtypes. In addition, the NeuroStem Chip can be used to characterize gene changes in intracerebral grafts of human cells, even when they are transplanted into experimental animals.

We specifically wish to stress that we are about to make the NeuroStem Chip available at a non-profit cost to the research community. We believe it has the potential to become an important screening tool in the expanding field of hSC studies in application to neurological/neurodegenerative disorders.

## Methods

### Human embryonic stem cell (hESC) cultures

Undifferentiated hESCs of SA02 (Sahlgrenska 2) line (Cellartis AB, Göteborg, Sweden; see NIH Human Embryonic Stem Cell Registry at [46]) were maintained over a monolayer of human "feeder cells" (hFCs; human foreskin fibroblasts, ATCC; cell line CCD-1112Sk). Feeder cells were grown in hFC medium (Iscove's modified Dulbecco's medium (IMDM) supplemented with 10% heat-inactivated FCS (Stem Cell Technologies, USA) and 0.5% Penicillin/Streptomycin mix) for 11 passages. One day prior to hESC plating, hFC medium was washed away from the hFCs, the latter were resuspended in a hESC proliferation medium (VitroHES media (Vitrolife AB, Sweden) supplemented with 4 ng/ml human recombinant basic FGF (hrbFGF, Biosource International, USA) and plated in a central ring of gelatinized in vitro fertilization (IVF) dishes with a cell density of 120,000 cells/dish. The outer rings of the IVF dishes were filled with Dulbecco's modified Eagle medium (DMEM) supplemented with 0.5% Penicillin/Streptomycin mix. One half of the culture medium was replaced every other day. The cells were maintained at 37°C, 5% CO_2_, 95% humidity settings. Every 6 days, fragments of the hESC colonies (around 10–14 colonies per dish, measuring around 0.015 × 0.015 mm) that had an unaltered morphology (indicating lack of spontaneous differentiation) were mechanically cut from dishes using stem cell knives/transfer pipettes (SweMed Lab International AB, Sweden) and then plated on fresh hFCs.

### Commitment of hESCs towards neuronal/dopaminergic differentiation pathway

Co-culturing with the PA6 stromal cell line (MC3T3-G2/Pa6, from RIKEN Cell Bank Japan (RCB 1127), derived from newborn mouse calvaria rapidly generates high numbers of DA neurons from mouse and monkey ESCs by an unknown mechanism named stromal-derived inducing activity (SDIA; [28,29]). For differentiation experiments, PA6 cells were plated on gelatine-coated T25 flasks at 16 × 10^3 ^cells/cm^2 ^(400,000 cells/flask) density 2 days prior to introducing hESCs into the co-culture and cultured at PA6 culturing media (containing minimum essential medium alpha (α-MEM) supplemented with 10% FCS and 0.5% Penicillin/Streptomycin). Alternatively, PA6 cells were plated over Type I collagen-coated glass cover-slips placed in wells of 4-well-plates (50,000 cells/well, for immunocytochemical (ICC) analysis). Three hours prior to initiation of co-culture, PA6 cells were rinsed 3 times with PBS and media was replaced with co-culture media (Glasgow's modified Eagle's media (G-MEM) supplemented with 8% knock-out serum replacement (KSR), 2 mM glutamine, 0.1 mM non-essential amino-acids (NEAA), 1 mM pyruvate, 0.1 mM β-mercaptoethanol (βME) and 4 ng/μl bFGF). Fragments of hESC colonies (80–90 per flask; 4–5 per well of 4-well-plate) presenting undifferentiated morphology were manually passaged onto the confluent PA6 monolayer and cell co-cultures were maintained at 37°C, 5% CO_2_, 95% humidity settings. One half of the co-culture medium was replaced every other day for first 10 days, and daily onwards.

### Characterization of hESCs and hESC-derived cells by immunocytochemistry (ICC) and RT-PCR

IVF dishes with hESCs grown atop hFCs and 4-well plate dishes with hESCs growing atop PA6 cells were fixed with 4% paraformaldehyde (PFA) for 15 minutes at the day of passage/harvest (Day 6 of hESC/hFC co-culturing) and Day 16 of co-culturing with PA6 cells, respectively. Cells were pre-incubated with blocking solution containing PBS, 0.5% Triton X-100 and 5% of donkey serum. They were then incubated with primary antibodies in blocking solution overnight at room temperature. After three washes with PBS, cells were incubated with the donkey anti-rabbit IgG conjugated with FITC or anti-mouse Cy3 (1:200, Jackson ImmunoResearch Laboratories). Cells were then washed once with PBS, incubated with 1:1000 DAPI in PBS for 10 minutes, followed by another wash with PBS. Coverslips were mounted onto glass slides with PVA mounting medium containing anti-fading reagent DABCO. The following primary antibodies were used: mouse anti-Oct3/4 (1:500, Santa Cruz Biotechnology Inc.); rabbit anti-Ki67 (1:200, Novocastra Ltd.); rabbit anti-TH (1:500, Chemicon). Immunostained cell cultures were visualized using a Zeiss fluorescent microscope attached to a Nikon digital camera.

Using RT-PCR, all RNA samples used in this study were tested negative for the presence of gDNA (data not shown). The intron-spanning primers for RT-PCR were selected from published works or designed using Oligo 4.0 software (Molecular Biology Insight) or Clone Manager Suite 7.1 (Sci Ed Software, NC, USA) and ordered from TAG Copenhagen A/S, Denmark, as the following: Sox2, SRY-box 2: 5'-TAC CTC TTC CTC CC CTC CA-3', 5'-ACT CTC CTC TTT TGC ACC CC-3'; En1, Engrailed 1: 5'-AAG GGA CGA AAC TGC GAA CTC C-3', 5'-GAC ACG AAA GGA AAC ACA CAC TCT CG-3' [47]; Nanog: 5'-TGC TTA TTC AGG ACA GCC T-3', 5'-TCT GGT CTT CTG TTT CTT GAC T-3' [48]; Gapdh, glyceraldehydes-3-phosphate dehydrogenase: 5'-GGA AGG TGA AGG TCG GAG TCA A-3', 5'-GAT CTC GCT CCT GGA AGA TGG T-3'; Aldh1A1, aldehyde dehydrogenase 1 family, member A1: 5'-GGG CAG CCA TTT CTT CTC AC-3', 5'-CTT CTT AGC CCG CTC AAC AC-3' [49]; Sdha, succinate dehydrogenase: 5'-TGG GAA CAA GAG GGC ATC TG-3', 5'-CCA CCA CTG CAT CAA ATT CAT G-3' [50]; Tubb, β-tubulin: 5'-CTC ACA AGT ACG TGC CTC GAG-3', 5'-GCA CGA CGC TGA AGG TGT TCA-3'; Nestin: 5'-AGA GGG GAA TTC CTG CT GAG-3', 5'-CTG AGG ACC AGG ACT CTC TA-3' [47]; Actb, β-actin: 5'-CAT CGA GCA CGG CAT CGT CA-3', 5'-TAG CAC AGC CTG GAT AGC AAC-3' [51]; Th, Tyrosine hydroxylase: 5'-CGA GCT GTG AAG GTG TTT G-3', 5'-TTG GTG ACC AGG TGA TGA C-3'; Msx1, homolog of *Drosophila *muscle segment homolog 1: 5'-CTC AAG CTG CCA GAA GAT GC-3', 5'-TCC AGC TCT GCC TCT TGT AG-3'; Pitx2, paired-like homeodomain transcription factor 2: 5'-ACC TTA CGG AAG CCC GAG TC-3', 5'-TGG ATA GGG AGG CGG ATG TA-3' [49]. cDNA was synthesized from 1 mg of total RNA using SuperScript II (Invitrogen), and RT-PCR amplifications were performed using the MiniOpticon system (Bio-Rad) with REDTaq Polymerase (Sigma-Aldrich) essentially as described by the manufacturer. Following initial denaturation for 5 min at 95°C, DNA amplifications were performed for 35 (En1, Nanog, Aldh1a1), 33 (Sox2, Nestin, Th, Msx1), 32 (Tubb, Pitx2), 27 (Sdha), 25 (Actb) or 22 (Gapdh) cycles of 1 min at 95°C, 1 min at 55°C (En1, Pitx2), 57°C (Sox2, Nanog, Sdha, Nestin), 58°C (Tubb), 58.5°C (Aldh1a1, Th) or 59°C (Gapdh, Actb, Msx1), and 1 min at 72°C. The final extension was 5 min at 72°C. Twenty μl volumes of RT-PCR products were analyzed by electrophoresis at 1% agarose gels and visualized by ethidium bromide staining.

RNA purification and fluorescent dye incorporationFor RNA purification of undifferentiated hESCs, the latter were mechanically separated from hFCs, collected in a 500 μl volume of VitroHES media, rinsed in PBS buffer and spun down at 300 rcf for 5 min. hESC-derived cells grown atop PA6 cells were harvested using a papain dissociation kit (Worthington Biochemical Corporation), rinsed in PBS buffer and spun down as described above. The resulting cell pellets were resuspended in RLT buffer (Qiagen, USA), passed through the shredder column (Qiagen) and stored at -80°C until the RNA sample was purified following the RNeasy Micro Kit (Qiagen) protocol (without carrier RNA); with DNase I (Quiagen) treatment incorporated to the latter. RNA integrity was tested using both ND-1000 specrophotometer (NanoDrop, USA) and RNA Nano LabChip/2100 Bioanalyzer system (Agilent Technologies, USA).

Fluorescent label (24 nmol of the Cyanine 3-CTP (Cy3); PerkinElmer, USA) was incorporated to 350–500 ng of total RNA amplified using Low RNA Input Fluorescent Linear Amplification Kit (Agilent Technologies), generally following the kit manufacturer's protocol. Similarly, 24 nmols of the Cyanine 5-CTP (Cy5; PerkinElmer) fluorescent label were incorporated to 400 ng sample of Human Universal Reference RNA (Stratagene, USA); in addition, dye-swap replicate amplification were performed. Amplified fluorescent cRNA samples were purified using RNeasy mini-columns (Quiagen), and fluorescence of the eluted products was measured using ND-1000 specrophotometer (NanoDrop).

### Microarray technology

Long oligonucleotide probes (69–71 nucleotides) matching gene targets of interest were selected from Operon V2 and V3 human AROS sets (Operon Biotechnologies Inc., USA). Arrays were produced by the SweGene DNA Microarray Resource Centre, Department of Oncology at Lund University (Sweden) using a MicroGrid II 600R arrayer fitted with MicroSpot 10 K pins (Harvard BioRobotics, USA). Printing was performed in a temperature- (18–20°C) and humidity- (44–49% RH) controlled area on Corning UltraGAPS aminosilane slides (Corning Inc., USA) with 140 μm spot-to-spot centerdistance and 90–110 μm average spot size. Following printing, arrays were dried for 48 hours andstored in a dessicator until used. Microarray slides were UV-cross-linked (800 mJ/cm^2^), pre-hybridizedwith fluorescently labeled samples using the Pronto! Universal Microarray Hybridization Kit (Corning) and subsequently hybridized with test (Cy3-labeled)/reference (Cy5-labeled) RNA samples (or in reverse dye-labeling order) at 42°C for 17 h using a MAUI hybridization station (BioMicro Systems Inc., USA) and the Pronto! Universal Microarray Hybridization Kit, generally following manufacturer's instructions, with several minor adaptations [31].

### Data acquisition and statistical analysis

Immediately following the washing steps, the fluorescence intensities were measured using a confocal laser scanner (G2505B, Agilent Technologies). After image formatting by Tiff Image Channel Splitter Utility (Agilent Technologies) and grid annotation, a complete set of spots was visually inspected for each slide. Using GenePix Pro (Molecular Devices Corp. USA) flags for artifactual spots were annotated for each spot. Median pixel intensity minus the median local background for both dyes was used to obtain a test over reference intensity ratio. Data normalization was performed per array subgrid using LOWESS curve fitting with a smoothing factor of 0.33 [52,53]. All normalizations, filtering, merging of technical replicates and analyses were performed in the BioArray Software Environment database [32]. To visualize sample-dependent variation of spot intensities, data was uploaded to the TIGR MultiExperiment Viewer (MEV; [34]).

## Authors' contributions

Overall design of the project was performed by joint effort of all coauthors. S.V.A. developed the NeuroStem Chip design, participated in hESC growth and differentiation, and performed RNA sample purifications, fluorescent sample preparations, microarray hybridizations, microarray data formatting and RT-PCR experiments. N.S.C. performed all computer analysis of microarray data. A.S.C. participated in hESC growth and differentiation and performed extensive characterization of hESCs on all stages of differentiation protocol. All authors have contributed to the writing and approved the final manuscript.

## Supplementary Material

Additional file 1 Genes up-regulated in hESC population, as compared to human universal reference RNA. Lists 101 genes up-regulated in hESC cells, as compared to universal reference RNA sample; sorted based on average Log2 ratio. Click here for file

Additional file 2 Top 100 NeuroStem entries up-regulated in dopaminergic differentiation. Lists top 100 genes most up-regulated in hESC-derived cells, as compared to undifferentiated hESC sample; sorted based on average Log2 ratio. Click here for file

## Figures and Tables

**Table 1:** NeuroStem chip entries related to dopaminergic system.

Genes^a^	Function
Aadc, Ant2 (Slc25a5), Calb1, Dat (Slc6a3), Girk2 (Kcnj6), *Igf1*, *Ptx3*, *Th*	Markers of dopaminergic neurons
En1, En2, Lmx1b^b^, Pax5, Shh, *Wnt1*	Markers of dopaminergic progenitors
Adh2, *Lhx1 (Lim1)*, Lhx5 (Lim2)	Markers of dopaminergic progenitors maturation
*Drd2*^b^, Vmat2	Markers of mature dopaminergic neurons
*Gfra1*, Gfra2, Gfra3, Gfra4, Otx2	Markers of early dopaminergic neurons
Dlx1, Dlx2, Flj38973, Lmx1a	Related to differentiation of dopaminergic neurons
*Adcy7*, Alcam, Art (Artn), *Bcl11a*, Calca, Cart, Cbln1, Cdh2, *Col11a1*, Cx43, D1lip (Drd1ip), Darpp32 (Ppp1r1b), Dbh, *Doc2a*, Drd1, Drd3, Drd4, Drd5, *Drip78 (Dnajc14)*, Egln3 (Phd3), Egr1, Fos, Fxyd6, Galm, *Ghr*, Grin2c, Grp, Gsbs (C7orf16), Hs6st2, *Igfbp4*, Kcna5, *Kcnab1*, Math2, Mlp (Marcksl1), *Moxd1*, *Mpp3*, Nrip3, *Nrp2*, *Nt (Nts)*, *Ntn (Nrtn)*, Nurr1 (Nr4a2), Pac1 (Adcyap1r1), Pacap, *Plagl1 (Zac)*, Pvrl3 (Nectin 3), Rab3c, Rcn1, *Slc17a6 (Vglut2)*, Slc6a1, Sox6, Spp1, Tacr3, *Trhr*, Zfp161, Vav3, *Zdhhc2*	Related to dopaminergic system

The majority of genes listed may also possess other functions, not related to dopaminergic system. Four entries were omitted from the table, representing candidate markers of dopaminergic system now undergoing characterization (*unpublished data*).

^a ^Italics indicates genes up-regulated (Log2 ratio >1.0) in dopaminergic differentiation experiment. Rcn1, Log2 ratio >0.97; Fxyd6 and Zfp161, Log2 ratio >0.7).

^b ^May also possess other functions related to dopaminergic system.

**Table 2:** Selected categories of NeuroStem chip entries.

Category	Functional role	Examples
I. Stemness	Recognized markers of stemness	Oct3/4, Nanog, Tdgf1
	Candidate markers of stemness	Cpxm1, Hook1, Ddx21
	Germ cell markers	Rif1, Bnc1, Bnc2
	Hematopoietic stem cell markers	Hoxb4, Cdcp1, C1qr1
	Mesenchymal stem cell marker	Bmpr1a, Bmpr2, Cd49a
II. Proliferation	Proliferation markers	Ki67, Pcna, Myc
	Neural proliferation markers	Emx1, Gbx2
III. Development	Differentiation	Lifr, Ebaf, Lyar
	Neuronal differentiation	Ren, Rai1, Neurod2
	Dopaminergic differentiation	Dlx1, Dlx2, Lmx1a
	Neuronal maturation	Mecp2, Ebf3, Sox4
	Neuronal process formation	Hmgb1, Rage
	Axonal elongation and branching	Pi3, Map1b, Slit1
IV. Neural markers	Pan-neural markers	Gap43, Nfh, Eno2
	Markers of dopaminergic neurons	Th, Aadc, Dat
	Markers of cholinergic neurons	Ngf
	Markers of spinal neurons	Hoxc6
	Glial markers	S100β, Cd68
	Astrocyte markers	Gfap, Tapa1
	Oligodendrocyte markers	Mag, Mobp, Omg
V. Distinct markers	Normal tissues	
	Liver	Gata6, G6pd, Fabp1
	Pancreas	Tff3, Sst, Pax4
	Skeletal muscle	Itga7, Dmd, Tnnt3
	Cardiac muscle	Nkx2.5, Anf, Myhca
	Smooth muscle	Actg2, Cnn1, Sm22α
	Endothelial cells	Flt1, Vwf, Pecam1
	Blood cell subtypes	Cd4, Cd8, Cd19
	Cancer cells	Maspin
	Pancreatic cancer	Kras2
	Colon cancer	Mina53
	Breast cancer	Klk7
	Prostatic cancer	Hpn, Mat8
	Lung cancer	Rab5a, Tp63
	Ovarian cancer	Mgb2
	Hodgkin's lymphoma	Ptp4a, Atf5, p21snft
VI. Relevant groups	Apoptosis-related	p53, Psip1, Birc2
	Telomere-related	Tert, Terf2, Rap1
	Antioxidants	Sod1, Sod2, Gpx1
	Imprinted genes	Tseb3, Gnas1, Grb10
	FZD group	Fzd1, Fzd3, Sfrp1
	WNT group	Wnt1, Wnt7a, Wisp1
	BMP group	Bmp1, Bmp2, Bambi
	STAT group	Stat1, Stat3, Pias1
	FGF group	Fgf1, Fgf2, Fgfr4
	GDF group	Gdf2, Gdf3, Gdf9
	Caspases group	Casp1, Casp2, Hsp70
	Cyclins group	Ccna1, Ccnc, Cdk1
	Kruppel-like group	Klf2, Klf9, Znf184

Due to functional redundancy, individual genes may fit into more than one category.

**Table 3:** Pearson correlation coefficients of assay comparisons.

	hES (Cy3) : Ref (Cy5), 10:5 pmol	hES (Cy5) : Ref (Cy3), 10:5 pmol	hES (Cy3) : Ref (Cy5), 20:10 pmol
hES (Cy3) : Ref (Cy5), 10:5 pmol	X	X	X
hES (Cy5) : Ref (Cy3), 10:5 pmol	0.787	X	X
hES (Cy3) : Ref (Cy5), 20:10 pmol	0.966	0.821	X
hES (Cy5) : Ref (Cy3), 20:10 pmol	0.816	0.961	0.846

Ref, Human Universal Reference RNA (Stratagene, USA).

**Table 4:** Top 25 genes up-regulated in hESC population, as compared to human universal reference RNA.

N	Gene index^a^	Gene name	Log2 Ratio	S.E.M (%)	Spot Error
1.	*Hesx1*	Homeo box expressed in ES cells 1	4.11	5.76	0.0575
2.	*Grem1*	Gremlin 1 homolog, cysteine knot superfamily	3.86	7.14	0.1012
3.	*Cdh6*	Cadherin 6 (K-cadherin)	3.49	3.88	0.0186
4.	Gja1	Gap junction protein, α1 (connexin 43)	3.49	7.10	0.0485
5.	*Htr2b*	5-hydroxytryptamine (serotonin) receptor 2B	3.45	8.91	0.0655
6.	*Emx2*	Empty spiracles homolog 2 (Drosophila)	3.24	6.10	0.1202
7.	Epo	Erythropoietin	3.22	9.67	0.0407
8.	Fzd7	Frizzled homolog 7 (Drosophila)	3.08	4.99	0.0317
9.	*Kal1*	Kallmann syndrome 1 sequence	3.05	3.10	0.0684
10.	*Auts2*	Autism susceptibility candidate 2	3.03	7.49	0.0088
11.	*Zic3*	Zic family member 3 heterotaxy 1	2.98	5.59	0.0709
12.	Rds	Retinal degeneration, slow	2.95	10.00	0.0756
13.	*Ddit4l*	DNA-damage-inducible transcript 4-like	2.90	4.90	0.0357
14.	Crispld1	Cysteine-rich secretory protein LCCL domain containing 1 (LOC83690)	2.87	5.04	0.0990
15.	Lrrn1	Leucine rich repeat neuronal 1	2.74	3.94	0.1002
16.	Gpr23	G protein-coupled receptor 23	2.74	6.02	0.0702
17.	Mgc16186	Hypothetical protein MGC16186	2.70	3.50	0.1592
18.	Par1	Prader-Willi/Angelman region-1	2.69	18.44	0.0953
19.	Nap1l3	Nucleosome assembly protein 1-like 3	2.69	4.00	0.0398
20.	Cecr2	Cat eye syndrome critical region protein 2	2.65	6.40	0.0082
21.	Fgfr2	Fibroblast growth factor receptor 2	2.64	5.11	0.0545
22.	*Klkb1*	Kallikrein B, plasma (Fletcher factor) 1	2.63	9.96	0.0255
23.	*Calb1*	Calbindin 1, 28kDa	2.61	1.45	0.0719
24.	Fbn3	Fibrillin 3	2.60	12.68	0.0462
25.	Id4	Inhibitor of DNA binding 4	2.50	6.44	0.0467

Only entries with spot intensity in hESC sample >100 are shown. Entries are sorted based on average Log2 ratio (after filtering for expression in all 4 technical replicates). S.E.M., standard error of the mean expressed as percentage of the mean; Spot Error, estimate of the average measurement error for all spots for the given reporter across the 4 technical replicates.

^a ^Italics indicates genes down-regulated (Log2 ratio < -1.0) in dopaminergic differentiation experiment.

**Table 5:** Embryonic stem cell marker genes expressed in hES SA02 cell line, as detected by the NeuroStem Chip analysis

N	Gene index^a^	Gene name	Log2 Ratio	S.E.M (%)
1.	*Hesx1*	Homeo box expressed in ES cells 1	4.11	5.76
2.	*Grem1*	Gremlin 1 homolog, cysteine knot superfamily	3.86	7.14
3.	Gja1	Gap junction protein, α1 (connexin 43)	3.49	7.10
4.	*Zic3*	Zic family member 3 heterotaxy 1	2.98	5.59
5.	*Dnmt3b*	DNA (cytosine-5)-methyltransferase 3β	2.26	6.25
6.	*Sfrp1*	Secreted frizzled-related protein 1	2.01	8.80
7.	Kiaa1573	KIAA1573 protein	1.98	2.48
8.	*Dppa4*	Developmental pluripotency associated 4	1.97	2.80
9.	*Sox2*	SRY (sex determining region Y)-box 2	1.86	12.44
10.	*Foxo1a*	Forkhead box O1A (rhabdomyosarcoma)	1.85	9.15
11.	Hmgb1	High-mobility group box 1	1.84	1.71
12.	Ctbp2	C-terminal binding protein 2	1.77	9.16
13.	*Lin28*	Lin-28 homolog (C. elegans)	1.52	20.32
14.	*Utf1*	Undifferentiated embryonic cell transcription factor	1.21	9.28
15.	Lefty1	Left-right determination factor 1	1.06	8.72

Only entries with spot intensity in hESC sample >100 and Log2 ratio >1 are shown. Entries are sorted based on average Log2 ratio (after filtering for expression in all 4 technical replicates). S.E.M., standard error of the mean expressed as percentage of the mean.

^a ^Italics indicates genes down-regulated (< -1.0 Log2 ratio) in dopaminergic differentiation experiment. Nanog is down-regulated with Log2 ratio < -0.6.
