# Supplementary material for: "NeuroStem Chip": a novel highly specialized tool to study neural differentiation pathways in human stem cells
Source: BMC Genomics. 2007 Feb 8;8:46. doi: 10.1186/1471-2164-8-46 (PMC1802744; doi:10.1186/1471-2164-8-46)
Supplement: Additional file 1 — Genes up-regulated in hESC population, as compared to human universal reference RNA. Lists 101 genes up-regulated in hESC cells, as compared to universal reference RNA sample; sorted based on average Log2 ratio. [file 1471-2164-8-46-S1.doc]

Additional file 1. Genes up-regulated in hESC population,

as compared to human universal reference RNA.

| N | Gene index | | Gene name | Log2  Ratio | S.E.M. | S.E.M.  (%) |
| --- | --- | --- | --- | --- | --- | --- |
| 1. | Hesx1 | | Homeo box expressed in ES cells 1 | 4.11 | 0.237 | 5.76 |
| 2. | Grem1 | | Gremlin 1 homolog, cysteine knot superfamily | 3.86 | 0.276 | 7.14 |
| 3. | Cdh6 | | Cadherin 6 (K-cadherin) | 3.49 | 0.136 | 3.88 |
| 4. | Gja1 | | Gap junction protein, α1 (connexin 43) | 3.49 | 0.248 | 7.10 |
| 5. | Htr2b | | 5-hydroxytryptamine (serotonin) receptor 2B | 3.45 | 0.307 | 8.91 |
| 6. | Emx2 | | Empty spiracles homolog 2 (Drosophila) | 3.24 | 0.198 | 6.10 |
| 7. | Epo | | Erythropoietin | 3.22 | 0.311 | 9.67 |
| 8. | Fzd7 | | Frizzled homolog 7 (Drosophila) | 3.08 | 0.153 | 4.99 |
| 9. | Kal1 | | Kallmann syndrome 1 sequence | 3.05 | 0.095 | 3.10 |
| 10. | Auts2 | | Autism susceptibility candidate 2 | 3.03 | 0.227 | 7.49 |
| 11. | Zic3 | | Zic family member 3 heterotaxy 1 | 2.98 | 0.166 | 5.59 |
| 12. | Rds | | Retinal degeneration, slow | 2.95 | 0.295 | 10.00 |
| 13. | Ddit4l | | DNA-damage-inducible transcript 4-like | 2.90 | 0.142 | 4.90 |
| 14. | Crispld1 | | Cysteine-rich secretory protein LCCL domain containing 1 (LOC83690) | 2.87 | 0.145 | 5.04 |
| 15. | Lrrn1 | | Leucine rich repeat neuronal 1 | 2.74 | 0.108 | 3.94 |
| 16. | Gpr23 | | G protein-coupled receptor 23 | 2.74 | 0.165 | 6.02 |
| 17. | Mgc16186 | | Hypothetical protein MGC16186 | 2.70 | 0.094 | 3.50 |
| 18. | Par1 | | Prader-Willi/Angelman region-1 | 2.69 | 0.497 | 18.44 |
| 19. | Nap1l3 | | Nucleosome assembly protein 1-like 3 | 2.69 | 0.107 | 4.00 |
| 20. | Cecr2 | | Cat eye syndrome critical region protein 2 | 2.65 | 0.169 | 6.40 |
| 21. | Fgfr2 | | Fibroblast growth factor receptor 2 | 2.64 | 0.135 | 5.12 |
| 22. | Klkb1 | | Kallikrein B, plasma (Fletcher factor) 1 | 2.63 | 0.262 | 9.96 |
| 23. | Calb1 | | Calbindin 1, 28kDa | 2.61 | 0.038 | 1.45 |
| 24. | Fbn3 | | Fibrillin 3 | 2.60 | 0.329 | 12.68 |
| 25. | Id4 | | Inhibitor of DNA binding 4 | 2.50 | 0.161 | 6.44 |
| 26. | Cyp26a1 | | Cytochrome P450, family 26, subfamily A, polypeptide 1 | 2.49 | 0.357 | 14.35 |
| 27. | Fbn2 | | Fibrillin 2 (congenital contractural arachnodactyly) | 2.47 | 0.198 | 8.01 |
| 28. | Nap1l2 | | Nucleosome assembly protein 1-like 2 | 2.39 | 0.074 | 3.10 |
| 29. | Chst6 | | Carbohydrate (N-acetylglucosamine 6-O) sulfotransferase 6 | 2.32 | 0.141 | 6.08 |
| 30. | Fgf2 | | Fibroblast growth factor 2 (basic) | 2.28 | 0.057 | 2.49 |
| 31. | Dnmt3b | | DNA (cytosine-5-)-methyltransferase 3β | 2.26 | 0.142 | 6.25 |
| 32. | Lphn2 | | Latrophilin 2 | 2.25 | 0.111 | 4.92 |
| 33. | Trpc4 | | Transient receptor potential cation channel, subfamily C, member 4 | 2.25 | 0.107 | 4.75 |
| 34. | Rab3c | | RAB3C, member RAS oncogene family | 2.24 | 0.463 | 20.70 |
| 35. | Sall2 | | Sal-like 2 (Drosophila) | 2.22 | 0.129 | 5.79 |
| 36. | Atp8a1 | | ATPase, aminophospholipid transporter (APLT), Class I, type 8A, member 1 | 2.22 | 0.239 | 10.78 |
| Additional file 1. Genes up-regulated in hESC population,  as compared to human universal reference RNA (*Continued*). | | | | | | |
| N | Gene index | | Gene name | Log2  Ratio | S.E.M. | S.E.M.  (%) |
| 37. | Pgap1 | | GPI deacylase | 2.22 | 0.077 | 3.46 |
| 38. | Fxyd6 | | FXYD domain containing ion transport regulator 6 | 2.22 | 0.170 | 7.66 |
| 39. | Flj12644 | | Hypothetical protein FLJ12644 | 2.22 | 0.093 | 4.21 |
| 40. | Nell2 | | NEL-like 2 (chicken) | 2.21 | 0.219 | 9.92 |
| 41. | Nbea | | Neurobeachin | 2.20 | 0.232 | 10.54 |
| 42. | Runx1t1 | | Runt-related transcription factor 1; translocated to, 1 (cyclin D-related) | 2.18 | 0.049 | 2.24 |
| 43. | Flj10916 | | Hypothetical protein FLJ10916 | 2.18 | 0.038 | 1.75 |
| 44. | C2orf31 | | Chromosome 2 open reading frame 31 | 2.17 | 0.185 | 8.51 |
| 45. | Wasf3 | | WAS protein family, member 3 | 2.17 | 0.176 | 8.11 |
| 46. | Sox11 | | SRY (sex determining region Y)-box 11 | 2.17 | 0.290 | 13.38 |
| 47. | Rabgap1l | | RAB GTPase activating protein 1-like | 2.12 | 0.209 | 9.85 |
| 48. | Nlgn3 | | Neuroligin 3 | 2.11 | 0.090 | 4.27 |
| 49. | Ptprz1 | | Protein tyrosine phosphatase, receptor-type, Z polypeptide 1 | 2.10 | 0.245 | 11.70 |
| 50. | Sp8 | | Sp8 transcription factor | 2.06 | 0.029 | 1.39 |
| 51. | Gprc5b | | G protein-coupled receptor, family C, group 5, member B | 2.06 | 0.185 | 8.97 |
| 52. | Flj10884 | | Hypothetical protein FLJ10884 | 2.06 | 0.227 | 11.02 |
| 53. | Pvrl3 | | Poliovirus receptor-related 3 | 2.06 | 0.036 | 1.74 |
| 54. | Iqca | IQ motif containing with AAA domain | | 2.04 | 0.063 | 3.07 |
| 55. | Loc91461 | Hypothetical protein BC007901 | | 2.04 | 0.238 | 11.66 |
| 56. | Lrrn6a | Leucine rich repeat neuronal 6A | | 2.04 | 0.072 | 3.55 |
| 57. | Lrat | Lecithin retinol acyltransferase (phosphatidylcholine--retinol O-acyltransferase) | | 2.03 | 0.314 | 15.46 |
| 58. | Sfrp1 | Secreted frizzled-related protein 1 | | 2.01 | 0.177 | 8.80 |
| 59. | Mgc1136 | Hypothetical protein MGC1136 | | 2.00 | 0.040 | 2.02 |
| 60. | Flj32810 | Hypothetical protein FLJ32810 | | 2.00 | 0.192 | 9.62 |
| 61. | Slc39a10 | Solute carrier family 39 (zinc transporter), member 10 | | 1.99 | 0.063 | 3.14 |
| 62. | Kiaa1573 | KIAA1573 protein | | 1.98 | 0.049 | 2.48 |
| 63. | Fam46b | Family with sequence similarity 46, member B | | 1.97 | 0.270 | 13.66 |
| 64. | Dppa4 | Developmental pluripotency associated 4 | | 1.97 | 0.053 | 2.72 |
| 65. | Flj14001 | Hypothetical protein FLJ14001 | | 1.95 | 0.080 | 4.08 |
| 66. | Epha1 | EPH receptor A1 | | 1.95 | 0.133 | 6.83 |
| 67. | Kcnk12 | Potassium channel, subfamily K, member 12 | | 1.93 | 0.146 | 7.60 |
| 68. | Erbb2 | V-erb-b2 erythroblastic leukemia viral oncogene homolog 2, neuro/glioblastoma derived oncogene homolog (avian) | | 1.91 | 0.084 | 4.37 |
| 69. | Dpysl3 | Dihydropyrimidinase-like 3 | | 1.90 | 0.306 | 16.14 |
| Additional file 1. Genes up-regulated in hESC population,  as compared to human universal reference RNA (*Continued*). | | | | | | |
| N | Gene index | Gene name | | Log2  Ratio | S.E.M. | S.E.M.  (%) |
| 70. | Thbs4 | Thrombospondin 4 | | 1.88 | 0.231 | 12.29 |
| 71. | Gabrb3 | Gamma-aminobutyric acid (GABA) A receptor, β3 | | 1.88 | 0.102 | 5.44 |
| 72. | Fos | V-fos FBJ murine osteosarcoma viral oncogene homolog | | 1.87 | 0.053 | 2.85 |
| 73. | Naalad2 | N-acetylated α-linked acidic dipeptidase 2 | | 1.86 | 0.172 | 9.25 |
| 74. | Mapk10 | Mitogen-activated protein kinase 10 | | 1.86 | 0.414 | 22.29 |
| 75. | Sox2 | SRY (sex determining region Y)-box 2 | | 1.86 | 0.231 | 12.44 |
| 76. | Foxo1A | Forkhead box O1A (rhabdomyosarcoma) | | 1.85 | 0.170 | 9.15 |
| 77. | Ugp2 | UDP-glucose pyrophosphorylase 2 | | 1.85 | 0.131 | 7.06 |
| 78. | Hmgb1 | High-mobility group box 1 | | 1.84 | 0.032 | 1.71 |
| 79. | Loc113828 | Hypothetical protein BC011204 | | 1.83 | 0.078 | 4.27 |
| 80. | Rhou | Ras homolog gene family, member U | | 1.83 | 0.130 | 7.11 |
| 81. | Col1a1 | Collagen, type I, α1 | | 1.82 | 0.245 | 13.43 |
| 82. | Vtn | Vitronectin (serum spreading factor, somatomedin B, complement S-protein) | | 1.82 | 0.044 | 2.44 |
| 83. | Crym | Crystallin μ | | 1.81 | 0.348 | 19.19 |
| 84. | Ptbp2 | Polypyrimidine tract binding protein 2 | | 1.81 | 0.069 | 3.83 |
| 85. | Cyyr1 | Cysteine and tyrosine-rich 1 | | 1.81 | 0.148 | 8.22 |
| 86. | C6orf115 | Chromosome 6 open reading frame 115 | | 1.80 | 0.172 | 9.51 |
| 87. | Dusp4 | Dual specificity phosphatase 4 | | 1.80 | 0.260 | 14.45 |
| 88. | Ttc10 | Tetratricopeptide repeat domain 10 | | 1.80 | 0.082 | 4.54 |
| 89. | Ddit4l | DNA-damage-inducible transcript 4-like | | 1.79 | 0.361 | 20.19 |
| 90. | Frzb | Frizzled-related protein | | 1.79 | 0.486 | 27.19 |
| 91. | Gyltl1b | Glycosyltransferase-like 1B | | 1.78 | 0.082 | 4.60 |
| 92. | Fnbp1l | Formin binding protein 1-like | | 1.77 | 0.133 | 7.53 |
| 93. | Ctbp2 | C-terminal binding protein 2 | | 1.77 | 0.162 | 9.16 |
| 94. | Tyro3 | TYRO3 protein tyrosine kinase | | 1.74 | 0.049 | 2.80 |
| 95. | Aass | Aminoadipate-semialdehyde synthase | | 1.74 | 0.132 | 7.62 |
| 96. | Cutl2 | Cut-like 2 (Drosophila) | | 1.73 | 0.177 | 10.21 |
| 97. | Kif5c | Kinesin family member 5C | | 1.73 | 0.251 | 14.51 |
| 98. | C2orf31 | Chromosome 2 open reading frame 31 | | 1.72 | 0.076 | 4.43 |
| 99. | Gli2 | GLI-Kruppel family member GLI2 | | 1.72 | 0.259 | 15.03 |
| 100. | Magel2 | MAGE-like 2 | | 1.72 | 0.090 | 5.23 |
| 101. | Col1a2 | Collagen, type I, α2 | | 1.71 | 0.165 | 9.64 |

Only entries with intensity in hESC sample >100 are shown. Entries are sorted based on average Log2 ratio (after filtering for expression in all 4 technical replicates). S.E.M., standard error of the mean expressed as percentage of the mean.
