# Supplementary material for: "NeuroStem Chip": a novel highly specialized tool to study neural differentiation pathways in human stem cells
Source: BMC Genomics. 2007 Feb 8;8:46. doi: 10.1186/1471-2164-8-46 (PMC1802744; doi:10.1186/1471-2164-8-46)
Supplement: Additional file 2 — Top 100 NeuroStem entries up-regulated in dopaminergic differentiation. Lists top 100 genes most up-regulated in hESC-derived cells, as compared to undifferentiated hESC sample; sorted based on average Log2 ratio. [file 1471-2164-8-46-S2.doc]

Additional file 2. Top 100 NeuroStem entries

up-regulated in dopaminergic differentiation.

| N | Gene index | Gene name | Log2  Ratio |
| --- | --- | --- | --- |
| 1. | Fgf7 | Fibroblast growth factor 7 (keratinocyte growth factor) | 5.89 |
| 2. | Casp4 | Caspase 4, apoptosis-related cysteine peptidase | 5.04 |
| 3. | Ets1 | V-ets erythroblastosis virus E26 oncogene homolog 1 (avian) | 5.00 |
| 4. | Aph1a | Anterior pharynx defective 1 homolog A (C. elegans) | 5.00 |
| 5. | Pitx2 | Paired-like homeodomain transcription factor 2 | 4.84 |
| 6. | Gabra2 | Gamma-aminobutyric acid (GABA) A receptor, α2 | 4.67 |
| 7. | Phox2b | Paired-like homeobox 2b | 4.66 |
| 8. | Panx2 | Pannexin 2 | 4.64 |
| 9. | Ptx3 | Pentraxin-related gene, rapidly induced by IL-1 β | 4.58 |
| 10. | Cd93 | CD93 | 4.37 |
| 11. | Mef2c | MADS box transcription enhancer factor 2, polypeptide C (myocyte enhancer factor 2C) | 4.36 |
| 12. | Cga | Glycoprotein hormones, α polypeptide | 4.22 |
| 13. | Fabp1 | Fatty acid binding protein 1, liver | 4.19 |
| 14. | Gata6 | GATA binding protein 6 | 4.19 |
| 15. | St8sia1 | ST8 α-N-acetyl-neuraminide α-2,8-sialyltransferase 1 | 4.15 |
| 16. | Pelo | Pelota homolog (Drosophila) | 4.12 |
| 17. | Igf1 | Insulin-like growth factor 1 (somatomedin C) | 4.06 |
| 18. | Ntsr1 | Neurotensin receptor 1 (high affinity) | 4.04 |
| 19. | Robo2 | Roundabout, axon guidance receptor, homolog 2 (Drosophila) | 4.04 |
| 20. | Nkx2.5 | NK2 transcription factor related, locus 5 (Drosophila) | 4.00 |
| 21. | Plagl1 | Pleiomorphic adenoma gene-like 1 | 4.00 |
| 22. | Cdkn1c | Cyclin-dependent kinase inhibitor 1C (p57, Kip2) | 3.98 |
| 23. | Rarb | Retinoic acid receptor, β | 3.97 |
| 24. | Bche | Butyrylcholinesterase | 3.86 |
| 25. | Egr2 | Early growth response 2 (Krox-20 homolog, Drosophila) | 3.81 |
| 26. | Bmp5 | Bone morphogenetic protein 5 | 3.79 |
| 27. | Il6st | Interleukin 6 signal transducer (gp130, oncostatin M receptor) | 3.76 |
| 28. | Edn3 | Endothelin 3 | 3.73 |
| 29. | Gabrg2 | Gamma-aminobutyric acid (GABA) A receptor, γ2 | 3.70 |
| 30. | Sox17 | SRY (sex determining region Y)-box 17 | 3.68 |
| 31. | Bcl2 | B-cell CLL/lymphoma 2 | 3.67 |
| 32. | Dll4 | Delta-like 4 (Drosophila) | 3.61 |
| 33. | Reln | Reelin | 3.61 |
| 34. | Vcam1 | Vascular cell adhesion molecule 1 | 3.59 |
| 35. | Cdkn2a | Cyclin-dependent kinase inhibitor 2A (melanoma, p16, inhibits CDK4) | 3.58 |
| 36. | Cx3cr1 | Chemokine (C-X3-C motif) receptor 1 | 3.58 |
| 37. | Igf2as | Insulin-like growth factor 2 antisense | 3.55 |
| 38. | Syt4 | Synaptotagmin IV | 3.55 |
| 39. | Cias1 | Cold autoinflammatory syndrome 1 | 3.51 |
| 40. | Mdfi | MyoD family inhibitor | 3.49 |
| Additional file 2. Top 100 NeuroStem entries up-regulated in  dopaminergic differentiation *(Continued)*. | | | |
| N | Gene index | Gene name | Log2  Ratio |
| 41. | Slc17a6 | Solute carrier family 17 (sodium-dependent inorganic phosphate cotransporter), member 6 | 3.48 |
| 42. | Col11a1 | Collagen, type XI, α1 | 3.47 |
| 43. | Flt1 | Fms-related tyrosine kinase 1 (vascular endothelial growth factor/vascular permeability factor receptor) | 3.44 |
| 44. | Gabrb1 | Gamma-aminobutyric acid (GABA) A receptor, β1 | 3.44 |
| 45. | Nedd4 | Neural precursor cell expressed, developmentally down-regulated 4 | 3.43 |
| 46. | Crh | Corticotropin releasing hormone | 3.41 |
| 47. | Znf25 | Zinc finger protein 25 | 3.40 |
| 48. | Afp | Alpha-fetoprotein | 3.38 |
| 49. | Adam9 | ADAM metallopeptidase domain 9 (meltrin γ) | 3.33 |
| 50. | Ngfr | Nerve growth factor receptor (TNFR superfamily, member 16) | 3.33 |
| 51. | Pkhd1l1 | Polycystic kidney and hepatic disease 1 (autosomal recessive)-like 1 | 3.30 |
| 52. | Itga4 | Integrin, α4 (antigen CD49D, α4 subunit of VLA-4 receptor) | 3.28 |
| 53. | Hoxb6 | Homeobox B6 | 3.25 |
| 54. | Nrtn | Neurturin | 3.24 |
| 55. | Cdh5 | Cadherin 5, type 2, VE-cadherin (vascular epithelium) | 3.24 |
| 56. | Cd34 | CD34 | 3.23 |
| 57. | Fzd10 | Frizzled homolog 10 (Drosophila) | 3.23 |
| 58. | Wnt3 | Wingless-type MMTV integration site family, member 3 | 3.23 |
| 59. | Rcv1 | Recoverin | 3.20 |
| 60. | Dcn | Decorin | 3.19 |
| 61. | Il10ra | Interleukin 10 receptor, α | 3.17 |
| 62. | Bcl2l10 | BCL2-like 10 (apoptosis facilitator) | 3.15 |
| 63. | Tep1 | Telomerase-associated protein 1 | 3.15 |
| 64. | Pla2g4a | Phospholipase A2, group IVA (cytosolic, calcium-dependent) | 3.14 |
| 65. | Meis1 | Meis1, myeloid ecotropic viral integration site 1 homolog (mouse) | 3.14 |
| 66. | Nell1 | NEL-like 1 (chicken) | 3.13 |
| 67. | Gbx2 | Gastrulation brain homeobox 2 | 3.13 |
| 68. | Snap25 | Synaptosomal-associated protein, 25kDa | 3.12 |
| 69. | Fn1 | Fibronectin 1 | 3.12 |
| 70. | Nfia | Nuclear factor I/A | 3.12 |
| 71. | Tff2 | Trefoil factor 2 (spasmolytic protein 1) | 3.10 |
| 72. | Gng11 | Guanine nucleotide binding protein (G protein), γ11 | 3.10 |
| 73. | Fgf11 | Fibroblast growth factor 11 | 3.09 |
| 74. | Pdgfra | Platelet-derived growth factor receptor, α polypeptide | 3.07 |
| 75. | Terf2ip | Telomeric repeat binding factor 2, interacting protein | 3.07 |
| 76. | Fzd6 | Frizzled homolog 6 (Drosophila) | 3.06 |
| 77. | Dlk1 | Delta-like 1 homolog (Drosophila) | 3.06 |
| 78. | Bace1 | Beta-site APP-cleaving enzyme 1 | 3.06 |
| Additional file 2. Top 100 NeuroStem entries up-regulated in  dopaminergic differentiation *(Continued)*. | | | |
| N | Gene index | Gene name | Log2  Ratio |
| 79. | Cd14 | CD14 molecule | 3.05 |
| 80. | Wnt10b | Wingless-type MMTV integration site family, member 10B | 3.04 |
| 81. | Ctsz | Cathepsin Z | 3.02 |
| 82. | Vim | Vimentin | 3.02 |
| 83. | Msx1 | Msh homeobox homolog 1 (Drosophila) | 3.00 |
| 84. | Cspg4 | Chondroitin sulfate proteoglycan 4 (melanoma-associated) | 3.00 |
| 85. | Tcirg1 | T-cell, immune regulator 1, ATPase, H+ transporting, lysosomal V0 subunit A3 | 2.99 |
| 86. | Th | Tyrosine hydroxylase | 2.99 |
| 87. | Cdk6 | Cyclin-dependent kinase 6 | 2.94 |
| 88. | Sparcl1 | SPARC-like 1 (mast9, hevin) | 2.94 |
| 89. | Tgfb1 | Transforming growth factor, β1 (Camurati-Engelmann disease) | 2.94 |
| 90. | Csf1r | Colony stimulating factor 1 receptor, formerly McDonough feline sarcoma viral (v-fms) oncogene homolog | 2.94 |
| 91. | Cinp | Cyclin-dependent kinase 2-interacting protein | 2.93 |
| 92. | Ccng2 | Cyclin G2 | 2.92 |
| 93. | Bmp2 | Bone morphogenetic protein 2 | 2.91 |
| 94. | Gprc5a | G protein-coupled receptor, family C, group 5, member A | 2.91 |
| 95. | Znf117 | Zinc finger protein 117 (HPF9) | 2.91 |
| 96. | Wif1 | WNT inhibitory factor 1 | 2.91 |
| 97. | Nrp1 | Neuropilin 1 | 2.88 |
| 98. | Col1a2 | Collagen, type I, α2 | 2.88 |
| 99. | Vegf | Vascular endothelial growth factor | 2.88 |
| 100. | Lhx1 | LIM homeobox 1 | 2.86 |

Entries are sorted based on average Log2 ratio (after filtering for expression in all 4 technical replicates).
